# *Biatoraalnetorum* (Ramalinaceae, Lecanorales), a new lichen species from western North America

**DOI:** 10.3897/mycokeys.48.33001

**Published:** 2019-03-05

**Authors:** Stefan Ekman, Tor Tønsberg

**Affiliations:** 1 Museum of Evolution, Uppsala University, Norbyvägen 16, SE-752 36 Uppsala, Sweden Uppsala University Uppsala Sweden; 2 Department of Natural History, University Museum, University of Bergen, Allégaten 41, P.O. Box 7800, NO-5020 Bergen, Norway University of Bergen Bergen Norway

**Keywords:** *
Biatora flavopunctata
*, *
Biatora pallens
*, *
Lecania
*, BAli-Phy

## Abstract

*Biatoraalnetorum* S. Ekman & Tønsberg, a lichenised ascomycete in the family Ramalinaceae (Lecanorales, Lecanoromycetes), is described as new to science. It is distinct from other species of *Biatora* in the combination of mainly three-septate ascospores, a crustose thallus forming distinctly delimited soralia that develop by disintegration of convex pustules and the production of atranorin in the thallus and apothecia. The species is known from the Pacific Northwest of North America, where it inhabits the smooth bark of Alnusalnobetulasubsp.sinuata and *A.rubra*. *Biatoraalnetorum* is also a new host for the lichenicolous ascomycete *Sclerococcumtoensbergii* Diederich.

## Introduction

During field work in the Pacific Northwest of the United States and Canada in 1995–2018, the second author came across a distinct crustose and sorediate lichen on the smooth bark of alders. Ascospores produced in the scattered pale-coloured apothecia turned out to be mostly three-septate, which prompted a search amongst the many names once described in or combined into the genus *Bacidia* De Not. As we were unable to find any previous description of a species fitting this morphology, it is described here as new to science. Morphological characteristics, primarily the combination of the structure of the proper exciple, sorediate thallus and ascospore shape, led us to suspect the new species to be a member of the genus *Biatora* Fr. (Printzen 1995, 2014).

## Materials and methods

Microscopic quantitative characters were investigated either in a 10% aqueous solution of potassium hydroxide (KOH) (ascospores, paraphyses) or in pure water (all other microscopic characters). Pigments were investigated and characterised using a 10% aqueous solution of KOH and a 50% v/v aqueous solution of commercial-grade nitric acid (70% HNO_3_). Measurements of quantitative characters are given either as ‘minimum value – maximum value’ or ‘minimum value – arithmetic mean value – maximum value (s = sample standard deviation, n = sample size)’. Lichen substances were screened with Thin Layer Chromatography (TLC) in solvent systems A, B’ and C following Culberson and Kristinsson (1970), Culberson (1972) and Menlove (1974). Aluminium plates were used in systems A and B’ and glass plates in system C, the latter to allow the detection of fatty acids.

In order to obtain some indication of relationships from other than morphological data, we obtained a complete sequence from the internal transcribed spacer (ITS) region of the ribosomal DNA using the laboratory approach described by Ekman and Blaalid (2011). Subsequently, we downloaded the data (S15023) of Printzen (2014) from TreeBase (https://treebase.org) and excised the ITS region. For reasons of computational tractability, we removed *Cliostomumgriffithii* (Sm.) Coppins (shown by Kistenich et al. 2018 to be more closely related to *Ramalina* Ach.), *Mycobilimbiapilularis* (Hepp ex Körb.) Hafellner & Türk (the genus already being well represented by two other species), sequences not definitively referred to any taxon (marked “cf.”) and all but one sequence from taxa represented by multiple accessions. Question-mark symbols were either removed (when they were terminal) or replaced by “N” (when they were internal). Finally, all gaps were stripped. To this data, we added our own ITS sequence of *Biatoraalnetorum* (MH818375), generated from Tønsberg 27500 (BG), resulting in a dataset with 45 sequences. We carried out a joint estimation of alignment and phylogeny using BAli-Phy version 3.1.4 (Suchard and Redelings 2006). We set the substitution model to a single GTR+I+Γ (the gamma distribution divided into four categories) and the gap model to a single RS07 model (Redelings and Suchard 2007) without partitioning the data. Priors were kept at their default values. The analysis consisted of 10 parallel runs and included a pre-burn-in of 10 iterations followed by 75000 cycles of Markov chain Monte Carlo (MCMC), sampling states every 50 cycles. The first 25000 cycles of each run were removed as burn-in. A more precise estimate of the time to convergence was obtained with the *statreport* tool of BAli-Phy.

## Results

**Figure 1.**
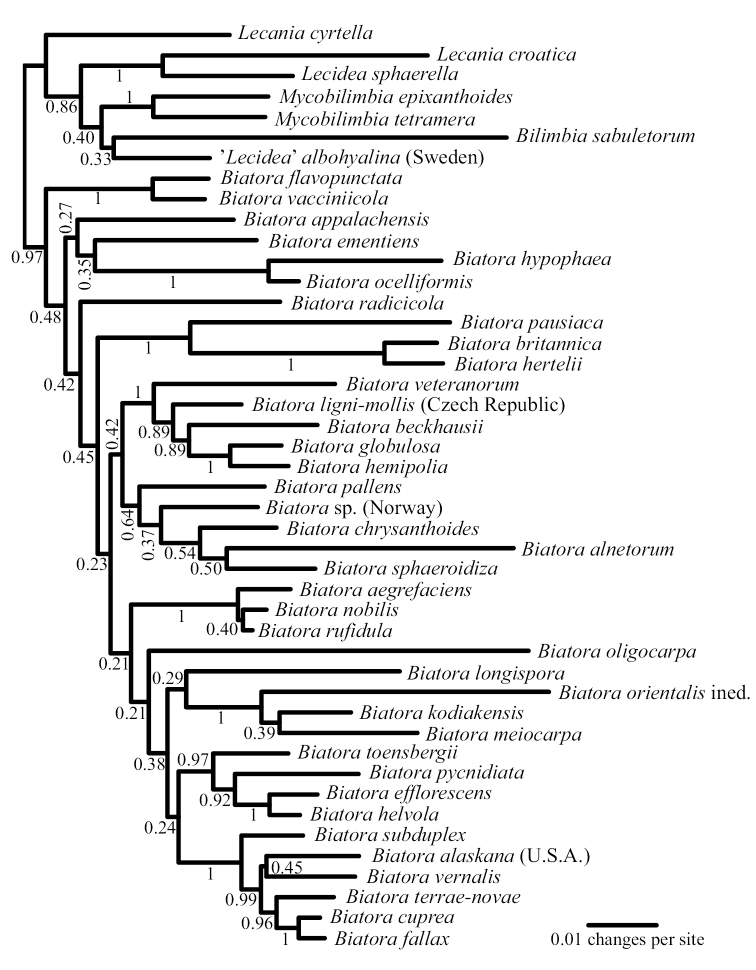
Majority-rule consensus tree of a Bayesian posterior sample obtained by joint estimation of alignment and phylogeny from ITS sequence data with BAli-Phy. The ingroup consists of the genus *Biatora* and the outgroup of members of *Lecanias. lat.*, *Mycobilimbia*, *Bilimbia* and ‘*Lecidea’ albohyalina*. Branch lengths are represented by their average across the posterior sample.

All numerical parameters of the BAli-Phy analysis had converged after 16650 cycles, but we anyway excluded the first 25000 cycles (resulting in a posterior sample of size 10×(75000-25000)/50 = 10000). In the posterior sample, the average standard deviation of split frequencies at or above 0.1 was 0.015. A majority-rule consensus tree with all compatible groups provided 0.97 posterior probability for the genus *Biatora*, including *B.alnetorum* (Fig. 1). In our consensus phylogeny, *B.alnetorum* appears in an unsupported clade (posterior probability 0.64) together with *B.chrysanthoides* Printzen & Tønsberg, *B.sphaeroidiza* Printzen & Holien, *B.pallens* (Kullh.) Printzen and an unnamed *Biatora* species from Norway.

### Taxonomy

#### Biatora alnetorum

Taxon classificationFungiLecanoralesRamalinaceae

S. Ekman & Tønsberg sp. nov.

829438

Figs 2 , 3 , 4

##### Diagnosis.

Similar to *Biatorapallens* (Kullh.) Printzen in having 3-septate ascospores and crystals in the exciple, but differs from that species primarily by the sorediate thallus, the production of atranorin in the thallus and proper exciple and in sometimes producing up to 7-septate ascospores.

##### Types.

U.S.A. Washington, Cowlitz Co., 7–8 km SW of summit of Mount St. Helens, E of Goat Mtn, NE of Goat Marsh Lake, N of Coldspring Creek, the W-facing slope W of gravel Rd FR 8123, 46°10'N, 122°17'W, elev. 900–1000 m, corticolous on Alnusalnobetulasubsp.sinuata, 8 Aug 1996, *T. Tønsberg 24071* (holotype: BG-L-101921; isotypes: BM, CANL, FR, NY, O, OSC, TRH, UPS, UBC, WTU).

##### Etymology.

The epithet, *alnetorum*, means ‘of the alder stands’ and is a reference to the fact that *Biatoraalnetorum* prefers thickets dominated by Alnusalnobetulasubsp.sinuata.

##### Description.

*Thallus* crustose, thin, continuous, finely cracked, whitish, forming ± convex pustules from which soralia develop. *Pustules* greenish (with no hint of yellow; pale grey in the herbarium), glossy, mostly discrete, firm, rounded or rarely ± ellipsoid in outline, 0.08–0.32 mm diam. when rounded and 0.12–0.60 × 0.10–0.36 mm diam. when ellipsoid. *Soralia* forming by disintegration of pustules into convex aggregations of yellowish grass-green (pale straw in the herbarium) and loosely arranged soredia, finally eroding into ± empty and shallow pits. *Soredia* mostly ellipsoid, rarely globose, strikingly similar in shape and size, firm (not easily disintegrating in squash preparations), 22–47–79 µm long (s = 14, n = 30) and 19–32–46 µm wide (s = 8, n = 30). *Prothallus* lacking. *Photobiont* unicellular, chlorococcoid, globose to (irregularly) ellipsoid, 7.5–12.5 µm long.

*Apothecia* absent or sparse, sometimes abundant, biatorine, 0.3–0.5–0.9 mm diam. (s = 0.1, n = 50), at first flat, later moderately convex, epruinose or thinly pruinose on edge. Disc pale pink (or pale yellowish with age in the herbarium). Margin pale pink to almost white, concolorous with disc or slightly paler, thick, distinct, raised above disc in young apothecia, soon level with the disc, persistent.

*Proper exciple* laterally 54–68 µm thick, with abundant minute crystals (< 1 µm diam.) that are soluble in KOH, colourless or diffusely pale orange-yellow, prosoplechtenchymatous, composed of radiating hyphae that branch in the inner but not outer part of the exciple, with gelatinised cell walls; cell lumina narrowly cylindrical, 0.7–0.8 µm wide (swelling in KOH); terminal cells not swollen or moderately swollen to 2 µm. *Hypothecium* colourless to pale orange-yellow, without crystals. *Hymenium* 41–56–63 µm thick (s = 6, n = 25), colourless or diffusely pale orange-yellow, usually without crystals, rarely with a thin and uneven layer of crystals at the surface. *Paraphyses* 1.6–2.1–2.8 µm wide in mid-hymenium (s = 0.3, n = 25), unbranched or moderately branched in upper part, sometimes sparingly anastomosed in lower part; apices not swollen to ± clavate, 1.6–2.8–4.7 µm wide (s = 0.6, n = 50), without internal pigment. *Asci* clavate, 8-spored; young spore mass forming a wide and bluntly conical ocular chamber, apex above young spore mass staining blue in IKI except for a pale blue and narrowly conical axial body surrounded by a dark blue zone (i.e. approximately of *Biatora*-type *sensu*Hafellner (1984)). *Ascospores* colourless, without perispore or ornamentation, bacilliform to short-acicular, straight or slightly curved to shallowly sigmoid, sometimes coiled in ascus, 17–30–53 µm long (s = 6, n = 50), 1.8–2.4–3.6 µm wide (s = 0.3, n = 50), 7.0–12.9–22.0 times as long as wide (s = 2.9, n = 50), mostly with 3 but sometimes with up to 7 septa.

*Pycnidia* not seen.

*Chemistry*: Large amounts of atranorin in thallus and apothecia. Thallus, soralia and proper exciple K+ yellow.

*Pigments*: No pigments or small amounts of Rubella-orange (Meyer and Printzen 2000) in proper exciple, hypothecium and/or hymenium.

**Figure 2.**
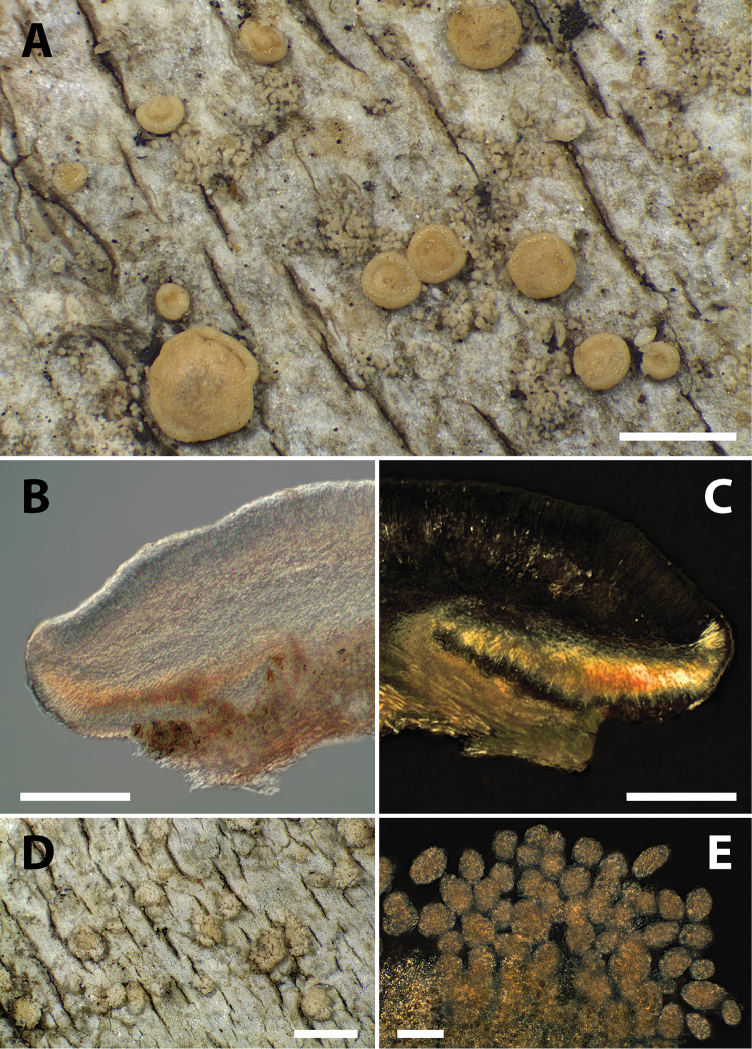
Morphology of *Biatoraalnetorum*. **A** Habit of lichen thallus with apothecia and soralia in herbarium specimen from 1999 (Tønsberg 27500, BG) **B, C** section through apothecium (Tønsberg 24077, BG), **B** showing pigmentation in bright-field illumination and **C** showing crystals in the proper exciple in cross-polarised light **D** thallus with soralia in herbarium specimen from 2000 (Tønsberg 28771a, BG) **E** soralium with soredia in cross-polarised light (Tønsberg 28771a, BG). Scale bars: 0.5 mm (**A**), 100 µm **(B, C**), 0.5 mm (**D**), 50 µm (**E**).

**Figure 3.**
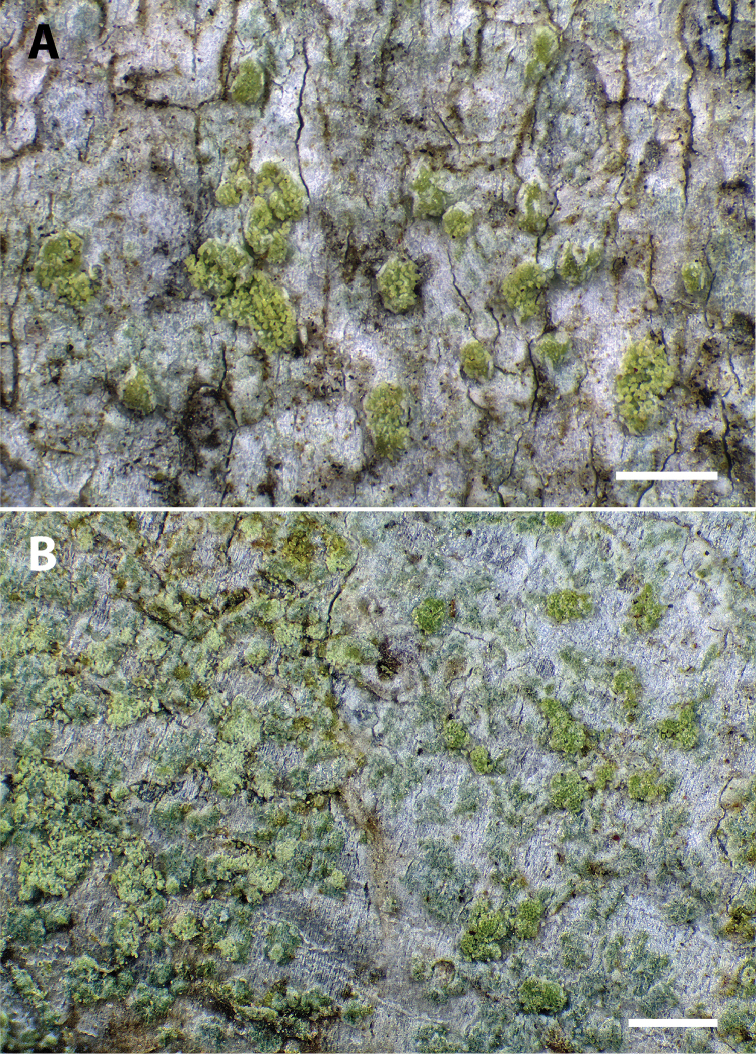
Morphology of *Biatoraalnetorum*. **A** Thallus with soralia in freshly collected specimen (Tønsberg 48200, UPS) **B** thalli with soralia in freshly collected specimens: *Biatoraalnetorum* to the right and the similar *B.flavopunctata* to the left (Tønsberg 48202, BG), separated more or less by the approximately vertical, shallow crack at the centre of the image. Scale bars: 0.5 mm (**A, B**).

**Figure 4.**
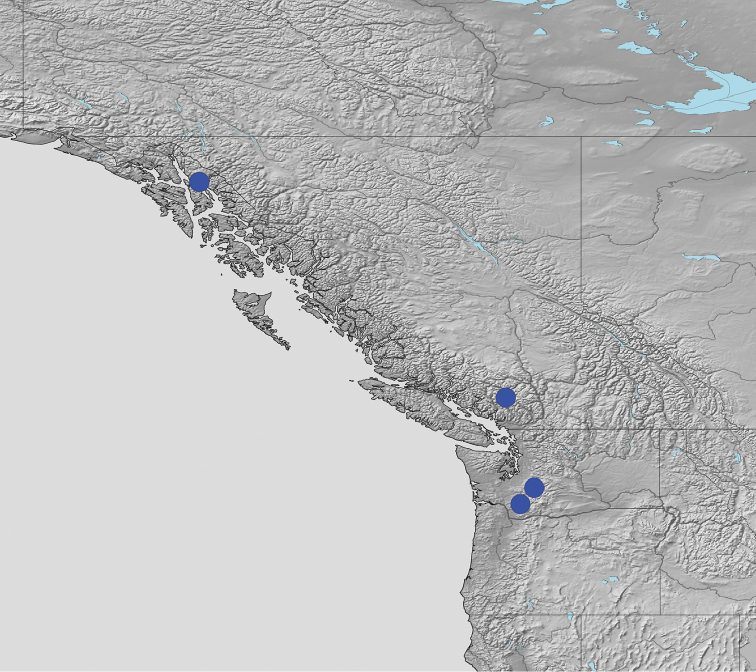
Known world distribution of *Biatoraalnetorum*, which includes the western United States and Canada.

##### Distribution and ecology.

*Biatoraalnetorum* is known from the Pacific Northwest of North America in Washington and Alaska (U.S.A.) and British Columbia (Canada). Its vertical distribution ranges from 620 to 1450 m a.s.l. It occurs in openings in humid old-growth coniferous forest and *Alnus* woodlands and in the alpine scrub zone. *B.alnetorum* inhabits smooth bark of trunks or, occasionally, branches. The phorophyte is almost exclusively Alnusalnobetulasubsp.sinuata (also known as Alnusviridissubsp.sinuata), except for a single record on *Alnusrubra*. Other lichens occurring together with *B.alnetorum* include (on *Alnusalnobetula*) *Caloplacasorocarpa* (Vain.) Zahlbr., *Biatoraflavopunctata* (Tønsberg) Hinter. & Printzen, *B.toensbergii* Holien & Printzen, *B.vacciniicola* (Tønsberg) Printzen and *Pertusariacarneopallida* (Nyl.) Anzi ex Nyl. and (on *Alnusrubra*) *Parmeliopsishyperopta* (Ach.) Arnold, *Japewiasubaurifera* Muhr & Tønsberg, and *Phlyctisspeirea* G. Merr.

##### Remarks.

Part of the type collection contains the lichenicolous fungus *Sclerococcumtoensbergii* Diederich (Diederich and van den Boom 2017). This fungus was, according to Diederich and van den Boom (2017) and Diederich et al. (2018), previously known to occur on *Megalariapulverea* (Borrer) Hafellner & E. Schreiner (Ramalinaceae) and *Pertusariacarneopallida* (Nyl.) Anzi ex Nyl. (Pertusariaceae). *Biatoraalnetorum* is reported here as a new host for this fungus.

##### Additional specimens examined.

Canada. British Columbia: N of Vancouver, Garibaldi Park, N of Wedgemount Creek, along Wedgemount Trail to Wedgemount Lake, 50°10.1'N, 122°50.2'W, elev. 1450 m, corticolous on horizontal trunks of Alnusalnobetulasubsp.sinuata over creek in old-growth coniferous forest, 25 Sept 2000, T. Tønsberg 28708 (BG, CANL, UBC). – U.S.A. Alaska: City and Borough of Juneau, along trail from Juneau to Mt. Robert, 58°17.8'N, 134°22.8'W, elev. 620–630 m, corticolous on ± horizontal trunks of Alnusalnobetulasubsp.sinuata, 7 Sept 1999, T. Tønsberg 27490 (BG); 58°17.7'N, 134°22.8'W, elev. 700 m, corticolous on ± horizontal trunks of Alnusalnobetulasubsp.sinuata, 7 Sept 1999, T. Tønsberg 27495 (BG); 58°17.7'N, 134°22.5'W, elev. 740–750 m, corticolous on ± horizontal, dead trunks of Alnusalnobetulasubsp.sinuata, 7 Sept 1999, T. Tønsberg 27497, 27499, 27500 (BG); 58°17.6'N, 134°22.2'W, elev. 800–850 m, corticolous on ± horizontal trunks of Alnusalnobetulasubsp.sinuata, 7 Sept 1999, T. Tønsberg 27501 (BG). Washington: Cowlitz Co., 7–8 km SW of summit of Mt St. Helens, E of Goat Mtn, NE of Goat Marsh Lake, N of Coldspring Creek, W of gravel Rd FR 8123, 46°10'N, 122°17'W, elev. 900–1000 m, corticolous on Alnusalnobetulasubsp.sinuata, 23 June 1995, T. Tønsberg 23432 (BG); corticolous on the underside of slightly leaning trunk of *Alnusrubra* in woodland with mature *Alnusrubra* and some large *Populus*, 23 June 1995, T. Tønsberg 23440 (BG); 8 Aug 1996, T. Tønsberg 24114 (BG); corticolous on Alnusalnobetulasubsp.sinuata in swamp, 8 Aug 1996, T. Tønsberg 24125 (BG); Lewis Co., Mount Rainier National Park, Nisqually River gorge, along Hwy 706, just E of the bridge, 46°46.9'N, 121°45.7'W, elev. 1165 m, corticolous on horizontal trunks of Alnusalnobetulasubsp.sinuata in NW-facing slope in river valley, 28 Sept 2000, T. Tønsberg 28771a (BG); 7 Oct 2018, T. Tønsberg 48200 & S. Bainbridge (BG, MORA, UPS, WTU); on branches of Alnusalnobetulasubsp.sinuata, 7 Oct 2018, T. Tønsberg 48201 & S. Bainbridge (BG, MORA, WTU); T. Tønsberg 48202 & S. Bainbridge (BG).

## Discussion

Morphologically, the new species stands out on account of its combination of pale pinkish apothecia with a proper exciple inspersed with small crystals, mostly 3-septate ascospores and green soralia formed from distinct pustules on the thallus and containing soredia that are remarkably even in size and shape. It agrees with other species of *Biatora* with regard to the radiating and (in lateral view) almost parallel excipular hyphae, the generally strongly gelatinised apothecial tissues, as well as the *Biatora*-type ascus (Hafellner 1984, Printzen 2014). Recent molecular phylogenies (e.g. Printzen 2014, Kistenich et al. 2018) have resulted in an increasingly inclusive delimitation of the genus compared to the first modern circumscription proposed by Printzen (1995). Concomitantly, the morphological amplitude accepted in the genus (tissue structures, chemistry, pigmentation etc.) has expanded. We adhere here to the most recent and quite broad circumscription of *Biatora* advocated by Printzen (2014) and confirmed by Kistenich et al. (2018) as monophyletic, the latter assuming that the genus *Myrionora* R. C. Harris is also included.

Our phylogeny (Fig. 1), which is based only on the internal transcribed spacer region, confirms that *B.alnetorum* is a member of the genus *Biatora* but does not allow any definitive conclusions beyond that. In the consensus tree, the new species appears as sister species to *B.sphaeroidiza* in a poorly supported group also including *B.chrysanthoides*, *B.pallens* and an undescribed species. Taking into account differences in taxon sampling, there are, however, different levels of support but no supported contradictions between our ITS phylogeny and the phylogeny of Printzen (2014), the latter based on more extensive DNA sequence data.

Amongst the previously known species of the genus, *Biatoraalnetorum* (Figs 2, 3) is morphologically most likely to be confused with the esorediate *B.pallens* and the sorediate *B.flavopunctata*. *B.pallens* and *B.alnetorum* share the 3-septate ascospores and the presence of crystals in the exciple. *B.alnetorum* is, however, different from that species in forming large, sorediate thalli, the occurrence of atranorin in the thallus and apothecia, larger apothecia that do not become as markedly convex and longer ascospores, with sometimes up to five or seven septa. In *B.pallens*, thalli are esorediate and often small, apothecia become convex with an excluded margin early during development and ascospores are consistently three-septate and short-bacilliform. Small amounts of usnic acid and zeorin occur in the thallus, whereas large amounts of usnic acid form crystals in the proper exciple and upper part of the hymenium (Ekman 1997). In the field, however, *Biatoraalnetorum* is most likely to be confused with *B.flavopunctata* (Fig. 3B), which is widespread on alpine shrubs in the Pacific Northwest. Both species are sorediate and often co-occur, forming mosaics on *Alnus* branches. In such situations, *B.alnetorum* is set apart by the presence of discrete and conspicuously yellowish grass-green soralia formed from convex pustules, whereas *B.flavopunctata* possesses pale (yellowish) green soralia not formed from convex pustules. Under the microscope, the soredia of *B.flavopunctata* are mostly globose and fragile, easily disintegrating in squash preparations. Ascospores are non-septate in *B.flavopunctata*(Tønsberg 1992; Printzen 1995) and chemical constituents include usnic, isousnic and often also stictic and cryptostictic acids in addition to atranorin (Tønsberg 1992, Printzen 1995). The anatomical and chemical differences between *B.flavopunctata* and *B.alnetorum* reflect the fact that the two species are unlikely to be closely related within the genus (Fig. 1). In addition, *B.alnetorum* shares the presence of punctiform soralia and more or less long-bacilliform, mainly three-septate ascospores with *B.bacidioides* Printzen & Tønsberg (Printzen and Tønsberg 2003). The latter, however, lacks crystals in the exciple, has near-black apothecia and produces argopsin, norargopsin and gyrophoric acid in the soralia.

The distribution of *Biatoraalnetorum* (Fig. 4) coincides with the Vancouverian Subprovince of the Cordilleran-Arctic Province of McLaughlin (2007). To our knowledge, it does not occur in the coastal or inland rain forest zones (Schoonmaker et al. 1997, Goward and Spribille 2005) but seems to prefer somewhat inland conditions. Having said that, *B.alnetorum* is, although unlikely to be common, probably overlooked and its distribution underestimated.

## Supplementary Material

XML Treatment for Biatora alnetorum
